# Correction: Tracking intramolecular energy redistribution dynamics in aryl halides: the effect of halide mass

**DOI:** 10.1039/c8ra90073f

**Published:** 2018-09-05

**Authors:** Xiaosong Liu, Yunfei Song, Wei Zhang, Gangbei Zhu, Zhe Lv, Weilong Liu, Yanqiang Yang

**Affiliations:** Department of Physics, Harbin Institute of Technology Harbin China; National Key Laboratory of Shock Wave and Detonation Physics, Institute of Fluid Physics, China Academy of Engineering Physics Mianyang China yqyang@caep.cn

## Abstract

Correction for ‘Tracking intramolecular energy redistribution dynamics in aryl halides: the effect of halide mass’ by Xiaosong Liu *et al.*, *RSC Adv.*, 2018, **8**, 29775–29780.

Incorrect corresponding authors were indicated in the published article while the correct version is shown above; Wei Zhang rather than Weilong Liu is the co-corresponding author.

The Royal Society of Chemistry apologises for these errors and any consequent inconvenience to authors and readers.

## Supplementary Material

